# Genetic Diversity and Ecosystem Functioning in the Face of Multiple Stressors

**DOI:** 10.1371/journal.pone.0045007

**Published:** 2012-09-18

**Authors:** Fabian Roger, Anna Godhe, Lars Gamfeldt

**Affiliations:** Department of Biological and Environmental Sciences, University of Gothenburg, Göteborg, Sweden; University of Zurich, Switzerland

## Abstract

Species diversity is important for a range of ecosystem processes and properties, including the resistance to single and multiple stressors. It has been suggested that genetic diversity may play a similar role, but empirical evidence is still relatively scarce. Here, we report the results of a microcosm experiment where four strains of the marine diatom *Skeletonema marinoi* were grown in monoculture and in mixture under a factorial combination of temperature and salinity stress. The strains differed in their susceptibility to the two stressors and no strain was able to survive both stressors simultaneously. Strong competition between the genotypes resulted in the dominance of one strain under both control and salinity stress conditions. The overall productivity of the mixture, however, was not related to the dominance of this strain, but was instead dependent on the treatment; under control conditions we observed a positive effect of genetic richness, whereas a negative effect was observed in the stress treatments. This suggests that interactions among the strains can be both positive and negative, depending on the abiotic environment. Our results provide additional evidence that the biodiversity-ecosystem functioning relationship is also relevant at the level of genetic diversity.

## Introduction

### Genetic Diversity and Ecosystem Functioning

The loss of species and its consequences for ecosystem functioning has received considerable attention in the last two decades [1], [2]. Species diversity has been shown to affect a range of ecosystem functions such as productivity, nutrient uptake efficiency, and decomposition [2], [3], as well as the stability of these same functions [4]. Genetic diversity, in turn, is a subject of great interest in the field of conservation biology [5] and agronomy [6], [7], but our understanding of the ecological importance of genetic diversity is still limited [2], [8]. It has been argued that differences at the genetic level can influence ecosystem processes [9] and that genetic diversity may play a role similar to that of species diversity in ecosystems with one or few numerically abundant key species [10]. Correspondingly, a growing body of research suggests that, like species diversity, genetic diversity influences a range of ecosystem processes and properties [10]–[19].

The underlying mechanisms by which genetic diversity may alter ecosystem processes are analogous to those proposed for species diversity. In both cases, the effects of diversity can be partitioned into ‘selection’ and ‘complementarity’ effects [20] where a selection effect occurs if the community includes a genotype with a specific trait that becomes dominant over time. The performance of the mixture is therefore determined by the performance of this genotype. Selection effects depend on the performance of each community member in monoculture and its relative abundance in the mixture but do not take into account any interaction between the community members. Complementarity effects occur when functioning increases or decreases as a result of interactions among the members. Examples of positive complementarity effects are ecological facilitation (i.e. mutualism and commensalism) and resource partitioning, and examples of negative complementarity effects are interference and exploitation competition. Selection effects can result in higher or lower functioning than expected based on the average performance of the genotypes in monoculture, which is called non-transgressive over-yielding. Complementarity effects can result in a diverse assemblage performing better than its best performing member, which is called transgressive over-yielding [21].

As diversity in general, genetic diversity may play a particularly important role in the face of multiple stressors. If different genotypes differ in their ability to deal with certain stressors, the capacity of a community to withstand a stressor depends on the presence of a resistant genotype. Genotypic richness increases the probability of the presence of such a genotype, and hence the capacity to uphold ecosystem functioning under stress conditions [11]. If the community is subject to multiple stressors occurring independently or simultaneously, genotypes that are resistant to each of the individual stressors or the combination of stressors are needed in order to sustain functioning. For this reason the selection effect of diversity can have a positive effect by itself, without including other effects such as positive complementarity [22]. In this study, we investigated the importance of genetic diversity for the growth of diatom cultures under temperature and salinity stress, using the marine diatom *Skeletonema marinoi* as a model organism.

### The Model System

*Skeletonema marinoi* Sarno & Zingone, 2005 (formerly *S. costatum*, [23]) is a common marine diatom species in temperate waters [24]. *S. marinoi* reproduces mainly asexually with growth rates of approximately one division per day. It forms long, monoclonal chains and it is easy to isolate and maintain in culture [25]. The genetic diversity within the species is large and is reflected in phenotypical diversity; even seasonally separated genotypes (hereafter referred to as strains) are found to differ in biovolume and growth rate [26]. The records of differentiated genetic populations and phenotypic variations make *S. marinoi* a highly suitable model species for the study of genetic diversity [27] and the short generation time permits the examination of changes in the community structure in laboratory experiments.

We used four genetically distinct strains of *S. marinoi* previously genotyped with eight polymorphic microsatellite loci, and cultured them both separately and together under control conditions, salinity stress, temperature stress and combined salinity and temperature stress. We defined stress as *“[…] external constraints limiting the rate of resource acquisition, growth or reproduction of organisms […]”* [28] which last over time, and we fixed the levels of salinity and temperature based on this definition. We characterised the strains by their maximum growth rates and biomass. In order to assess changes in the relative frequencies of the strains, we determined the clonal composition at the end of the experiment.

In accordance with the theoretical considerations made above, we developed four hypotheses. Our first hypothesis (hypothesis 1) was that both stressors impair community growth rates and limit standing stock biomass, and that the combination of these stressors impairs growth more than each single stressor alone. This hypothesis is to some extent self-fulfilling by definition, as the salinity and temperature levels were chosen to have this very effect, but it represents an important pre-requisite for the following hypotheses. Second, we made the assumption that the strains were phenotypically different and hence hypothesised that the different monocultures would display distinct growth rates and maximum biomass (hypothesis 2a). Moreover, we hypothesised that the strains differ in their ability to deal with one or both stressors, which could possibly result in one strain having a higher tolerance to one stressor and another strain having a higher tolerance to the other stressor (hypothesis 2b). If hypothesis 2 is true, different scenarios are possible for the mixture. If the strains express the same phenotypes in the mixture as in the monocultures, we expect the most successful strain in monoculture to outgrow its competitors and increase its relative frequency in the mixture (positive selection effect, hypothesis 3). Consequently, the mixture productivity in this scenario should be close to the yield of the most productive monoculture and should exceed the average yield of the four monocultures. Finally, facilitation or resource partitioning can occur (positive complementarity effect, hypothesis 4). If not counteracted, positive complementarity should result in transgressive over-yielding. Hypotheses 3 and 4 are not mutually exclusive; both selection and complementarity effects can occur at the same time, possibly cancelling each other out.

## Materials and Methods

### Experimental Set-up

We studied the growth dynamics of four strains of the marine diatom *S. marinoi* in both monoculture and in a mixture containing all four strains. The strains were cultured in two temperatures (20°C, 27°C) and two salinity levels (25, 7). Levels were chosen to represent near-optimal (20°C, 25) and heavy-stress (27°C, 7) conditions, as verified in pilot experiments and in accordance with published data [29], [30]. The design was completely factorial using salinity (two levels), temperature (two levels) and strains (comprising the mixture, five levels) as factors, resulting in 20 different combinations, replicated four times.

The strains used in the experiment were isolated in autumn 2009 from germinated resting stages embedded in surface sediment that was collected off the Swedish west coast in May 2009. The procedures for germinating, isolating and establishing the monoclonal cultures are described in Härnström et al. [31]. The reference names, genotypes and access names are summarized in Table 1. Prior to the experiment, the strains were pre-adapted for 5 days to intermediate temperature and salinity conditions (T = 25°C, S = 15). The experiment ran for ten days in a climate chamber and samples were taken daily. Initial concentrations were 6000 cells ml^–1^ in monocultures, and 1500 cells ml^–1^ of each strain in the mixture. The total experimental volume was 40 ml. In order to avoid differences in the initial concentrations among treatments, we inoculated all monocultures from the same four exponentially growing stock cultures, and we inoculated the mixture from a stock mixture prepared earlier the same day. We calculated cell densities of the stock cultures using a Sedwick-Rafter chamber (1801–G20 Wildlife Supply Company, Yvlee, USA). A minimum of 900 cells were counted per culture to estimate the density.

**Table 1:** Summary of the four strains used in the present experiment.

Strain	Name in Exp	S.mar 1	S.mar 2	S.mar 6
Lys6 AAF	Strain 1	192/**222**	383/383	**344**/350
Lys6 Q	Strain 2	**194**/**202**	383/387	**334**/350
Lys6 S	Strain 3	192/192	383/**389**	**346**/**352**
V 8	Strain 4	**206**/**218**	**385**/387	**328**/**354**

Strain indicates the names by which the strain can be accessed at Göteborg University Marine Algal Culture Collection (GUMACC). The respective genotypes were identified based on three microsatellite loci (S.mar 1, 2, 6). Bold numbers indicate unique alleles.

### Culture Conditions

We cultured the cells in 50 ml Nunc Nunclon™^Δ^ EasYFlasks™ with a vent closure permitting gas exchange, at irradiance 70–90 µmol photons s^–1^ m^–2^ (measured at lid height and provided by fluorescence tubes, L36W/865 Lumilux® Cool Daylight, Osram GmbH, Augsburg, Germany), with a 12h:12h light-dark photoperiod. We prepared the growth medium with filtered seawater (S = 35) from the Sven Lovén Center for Marine Sciences at Kristineberg, and diluted it with Milli-Q water to the intended salinities. The water was autoclaved and enriched with nutrients according to the standard recipe for f/2 medium [32]. No additional nutrients were added during the experiment. We positioned the flasks in two identical 50 L water baths, so that the lower culture-containing part was fully immersed. Temperature was adjusted by setting the room temperature to 20°C and heating one water bath with two commercial aquarium heaters to 27°C. The water was mixed using two aquarium pumps, and temperature was monitored daily. Diatoms were resuspended daily by gently inverting the flasks. We controlled for bottle-effects by rearranging the flask positions daily, following a randomized schedule.

### Sampling and Biomass Estimation

Samples were taken daily in a random order and at approximately the same time of the day. We used fluorescence of chlorophyll *a* (Chl *a*) as a proxy for biomass, reported in raw fluorescence units. We tested the correlation between cell counts and fluorescence prior to the experiment (log-log linear model, r^2^ = 0.97, N = 20, p<0.001). Chl *a* was extracted by a whole water extraction method [33] modified after Kremp et al. [34]. 600 µl samples were diluted in 5.4 ml Ethanol (99.5%, v/v) using Brandt® soda glass test tubes (w/o rim, round bottom, Ø 12 mm, height = 100 mm, VWR int.). The samples were measured with a fluorometer (TD-700 Turner Design) at a 665 nm excitation wavelength after a 1 h extraction in the dark at room temperature. The fluorometer range was calibrated to cover cell densities up to 600 000 cells ml^–1^. If the fluorescence reached more than half the maximum of the set range, we took only 300 µl samples the following day, and diluted subsequently with 300 µl Milli-Q water.

### Isolation of Chains

To determine clonal composition in the early stationary phase, we isolated 40 chains per replicate from both the control and the low salinity treatments. It was not possible to isolate single chains from the high temperature and the double-stressed treatment. In the high temperature treatment the chains stuck together, making the isolation of single chains impossible, and in the double-stressed treatment no growth was observed. We isolated cells on day 7 and 9 from the control treatment and on day 8 and 9 in the low salinity treatment (30 chains per replicate on day 7 and 8 respectively and 10 additional chains per replicate from both treatments on day 9). In total we isolated 160 chains per treatment. The chains were isolated by micropipetting as described in Godhe and Härnström [26]. 290 out of the 320 isolated chains (90.6%) grew to sufficient densities for DNA extraction.

### DNA Extraction and Microsatellite Genotyping

The isolates were sufficiently dense after approximately two weeks and the total volume of the cultures (40 ml) was filtered trough 3-µm-pore-size filter (Ø 25mm, Versapore®-3000, Pall Cooperation). We put filters in Eppendorf tubes (1.5 ml, Eppendorf AG, Hamburg, Germany), kept them on ice immediately after filtration, and stored them at –20°C. DNA was extracted within two weeks after filtration. Genomic DNA extraction was performed following the CTAB extraction protocol [35]. DNA concentration and purity was measured with a spectrophotometer (Pharmacia Biotech Gene-Quant II, Buckinghamshire, UK). Samples with a 260nm/280nm absorbance ratio below 1.3 were further purified with an E.Z.N.A® Sp-Plant DNA-kit (Omega bio-tek) following the manufacturer’s instructions. PCR and fragment analyses were performed at the Genomics Core Facility, Sahlgrenska Academy, University of Gothenburg, following the previously described procedure [36]. We determined the allele sizes for the three microsatellite loci using GeneMapper (ABI Prism® GeneMapper™ Software Version 3.0). The unambiguous samples were assigned according to the known loci of the respective strains, and weighted with 1 (216 samples out of 290). Despite the effort to isolate single chains, the reading of the microsatellite loci showed the presence of two genotypes in 28 samples. These samples were split into two sub-samples each of which we assigned to one of the strains and weighted 0.5. A total of 46 samples gave ambiguous or no readings or were lost during DNA preparation and were thus discarded. We calculated clonal composition as the relative proportions of the four strains per replicate.

### Data Analysis

We fitted a logistic growth model of the form:
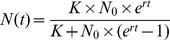
to the growth data of the four separate clones and of the mixture in the control, the high temperature and the low salinity treatments. In the fitted model, *N(t)* is the biomass of the population at time *t*, measured as raw fluorescence of *Chl a*; *t* is the time in days; *N_0_* is the biomass of the population at *t* = 0 which corresponded to 10 raw fluorescent units; *K* is the maximum biomass; and *r* is the maximum growth rate. Curve fitting was not possible in the double stressed treatment as all cells died. We fitted the model to the pooled data of the four replicates using the nonlinear least-square (*nls*) function in [R] [37]. Subsequently, *r* and *K* of each model fit were extracted and bootstrapped 100 000 times with the *rmvnorm ()* function and the variance and covariance estimates of the *nls ()* function. Finally, the density distribution of the bootstrapped growth parameters (*r, K*) was calculated with the *dmvnorm()* function and contour lines representing 95% confidence intervals were plotted. Two growth curves were assumed to be different when the corresponding confidence intervals did not overlap.

We used the growth parameter *K,* extracted form the logistic growth model, to calculate complementarity, selection and net biodiversity effects (see [20] for calculation details). This parameter represents the maximum biomass reached by each specific treatment and was taken as monoculture yield in the monocultures. The yield of each strain in the mixture was calculated by dividing the total observed yield (*K* of the mixtures) according to the relative proportions of the strains at the end of the experiment, determined by genotyping. Complementarity and selection could only be calculated for the two treatments where the clonal composition at the end of the experiment was known (*i.e.* control and low salinity,). To account for the uncertainty in the estimation of *K,* calculations were carried out for all pairs of bootstrapped *K*-values and 95% confidence intervals were computed.

We also investigated the combined effect of both stressors (*i.e.* low salinity and high temperature) to determine if this effect differed from what we expected based on the observations of the effects of each single stressor. We used the additive effect model that is consider to be the most appropriate when two stressors affect independent physiological processes [38], which was likely the case in this study. In the additive effect model the interaction between multiple stressors is called synergistic or antagonistic if the combined effect of all stressors on the considered variable is stronger or weaker, respectively, than expected for the sum of the effects of each stressor individually. If the combined effect is neither significantly weaker nor stronger, the effects are additive. We conducted the stress calculations for each clone and the mixture as described in Folt et al. [38] seperately for *r* and *K* based on the respective bootstrapped values. Next, we calculated the 95% confidence interval of the expected *r – K* distribution and compared it to the observed no-growth values in the combined stressor treatments. Accordingly, if the confidence interval included either *r* = 0 or *K* = 0, the effect was said to be additive. Synergism could not be assessed, however, as this would have meant that the observed values would be below 0.

We conducted the data analysis and graphical representation in [R] using the additional [R]-packages “mvtnorm” [39], “plyr” [40] and “ggplot2” [41].

## Results

**Figure 1:**
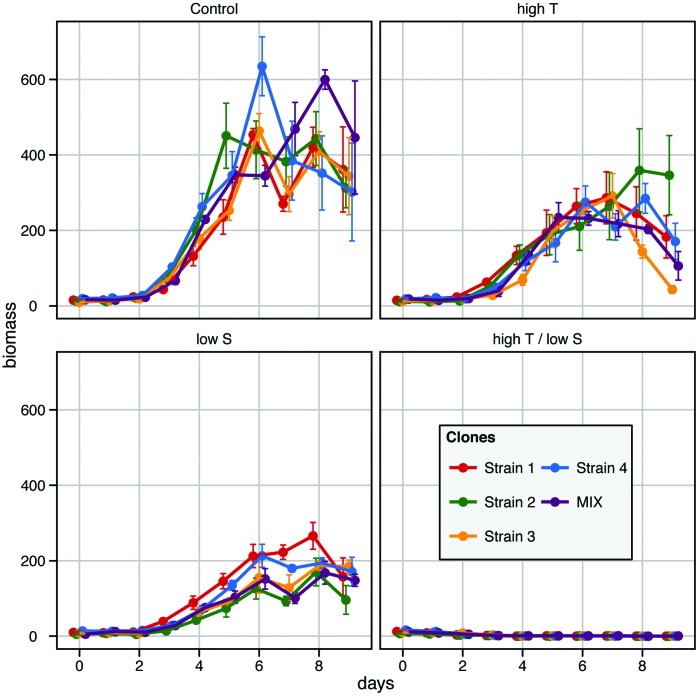
Growth curves. Growth curves of strain 1–4 (red, green, yellow and blue, respectively) and the mixture (violet) comprising all four clones in equal proportions. Each panel represents one salinity×temperature treatment (Control: Salinity 25, Temperature 20°C; high T: Salinity 25, Temperature 28°C; low S: Salinity 7, Temperature 20°C; high T/low S: Salinity 7, Temperature 28°C). Data points represent the mean of the four replicates; error bars represent standard error of mean. Start concentrations were 6000 cells ml^–1^ in all treatments. Biomass units are raw fluorescence data. The experiment was terminated on day 9.

Cells grew well in the control, the low salinity and the high temperature treatments but no growth was observed when both stressors were combined (Figure 1, Figure S1). Growth phases in the control and the two single-stressor treatments were similar, with the exponential growth phase starting on day 2 and lasting until day 6. All cultures started to decline on day 9, and hence the experiment was terminated on this day.

### Growth Dynamics

**Figure 2:**
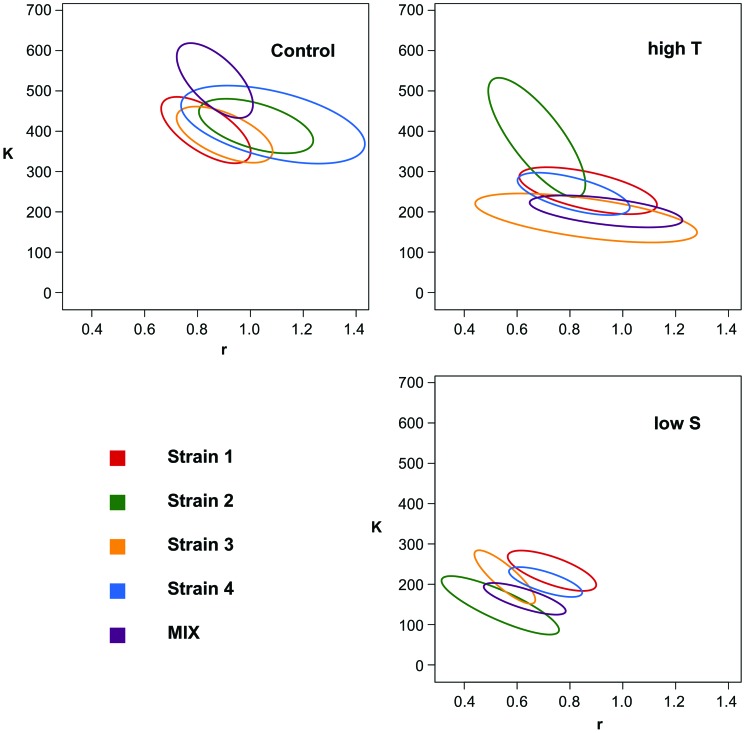
Growth dynamics. Growth dynamics of the four strains in monocultures (red, green, yellow and blue for strain 1–4 respectively) and the mixture (violet) in the three treatments in which growth was observed, plotted per treatment. Control: Temperature = 20°C, Salinity = 25; high T: Temperature = 28°C, Salinity = 27; low S: Temperature = 20°C, Salinity = 7. The ellipses represent 95% confidence intervals of the bootstrapped parameter distributions of the modelled growth curves. *K* represents maximum biomass and the units are raw fluorescence of Chl *a,* and *r* is the maximum growth rate given in cell divisions per day. The range of the axes is standardized in order to represent the same amount of relative variance around the overall mean of *r* and *K*, respectively.

**Figure 3:**
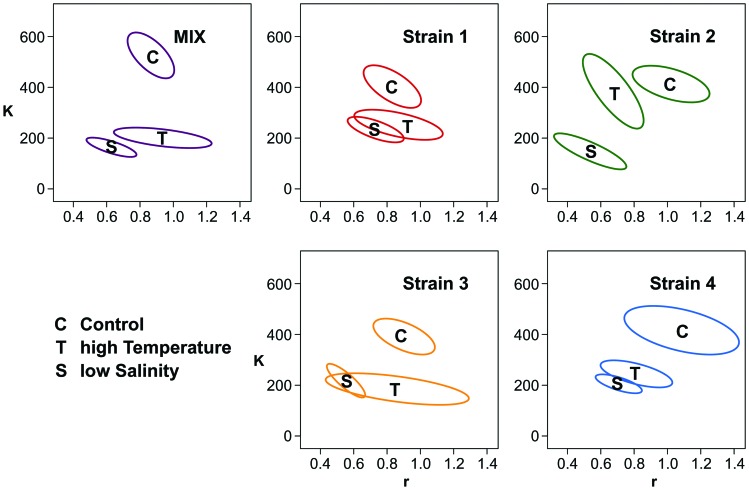
Growth dynamics. Growth dynamics of the four strains in monocultures (red, green, yellow and blue for clone 1–4 respectively) and mixture (violet) in the three treatments in which growth was observed, plotted per strain and the mixture. [C]: Temperature = 20°C, Salinity = 25; [T] = 28°C, Salinity = 27; [S]: Temperature = 20°C, Salinity = 7. The ellipses represent 95% confidence intervals of the bootstrapped parameter distributions of the modelled growth curves. *K* represents maximum biomass and the units are raw fluorescence of Chl *a*, and *r* is maximum growth rate given in cell divisions per day. The range of the axes is standardized in order to represent the same amount of relative variance around the overall mean of *r* and *K*, respectively.

On average, the *S. marinoi* cultures reached maximum cell densities and growth rates of roughly 1.8×10^5^ cells ml^–1^ and 0.94±0.1 divisions d^–1^ in the control treatment, 1×10^5^ cells ml^–1^ and 0.83±0.1 divisions d^–1^ in the high temperature treatment and 0.6×10^5^ cells ml^–1^ and 0.63±0.09 divisions d^–1^ in the low salinity treatment. Figure 2 & 3 present the growth characteristics of all strains, represented by the respective confidence interval of the bootstrapped *r*-*K* values. In both stressed treatments, all strains and the mixture showed different growth dynamics than in the control treatment (Figure 2). Interestingly, both dimensions (*r* and *K*) were needed to separate the growth curves in all treatments, highlighting the importance of assessing both parameters jointly. When we compared the growth curves of the strains within treatments, we observed no differences between strains in the control group, and only between strain 2 and strain 3 in the high temperature treatment. In the low salinity treatment, the growth curve of strain 2 differed from strain 1 and 4, and strain 3 differed from strain 1. When we compared the growth curves of each strain between the three treatments (Figure 3) we found differences between the low salinity and the high temperature treatment for strain 2, but not for strain 1, 3 and 4. These observations suggested that the strains did not grow differently *per se*, but that they reacted differently to the two stressors.

### Comparisons of the Mixture and Monocultures

The mixture did not consistently differ from the monocultures. In the control, the mixture showed higher growth than strain 1, and in the high temperature treatment it showed lower growth than strain 2. In the low salinity treatment the growth curves of the mixture showed lower growth than strain 1 and strain 4 (Figure 2).

**Figure 4:**
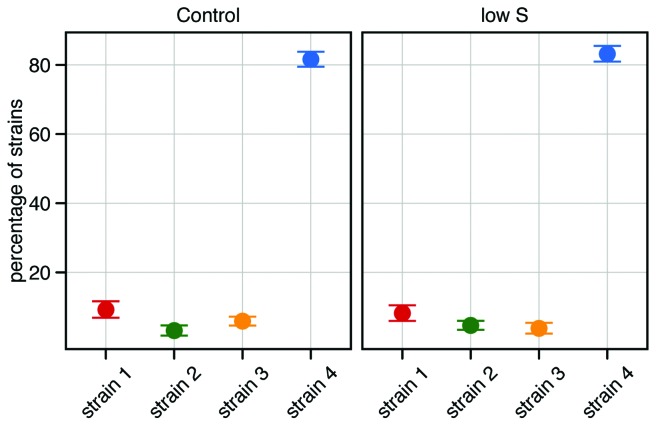
Relative abundances of the strains. Relative abundances of the strains 1–4 (red, green, yellow and blue, respectively) in the mixture of the control (Salinity 25, Temperature 20°C) and the low salinity (Salinity 7, Temperature 20°C) treatments. Proportions are given in percentages and represent the average abundance of the four replicates. Error bars are standard error of the mean.

We assessed the relative abundance of the strains in the early stationary growth phase in the control and the low salinity treatment. Strain 4 dominated the mixture in both treatments with average relative abundances of over 80% (Figure 4). This dominance was consistent throughout all the replicates, with a minimum abundance of 78%. We observed no differences between the abundances of strain 1, 2 and 3 (one factor ANOVA with “strain” as factor and a subsequent post-hoc Tukey HSD test, *P>*0.15 in all cases).

### Stress and Biodiversity Calculations

It was not possible to assess synergistic effects of the combined stress treatment as the 95% confidence intervals of the expected distributions included or were below 0 in all cases. Although this overlap was primarily observed for the *K*-axis, *K* and *r* cannot be considered independent variables, and an independent interpretation of *r* would be invalid. Despite this, we were able to determine that the combined effect of both stressors was additive at a minimum.

**Figure 5:**
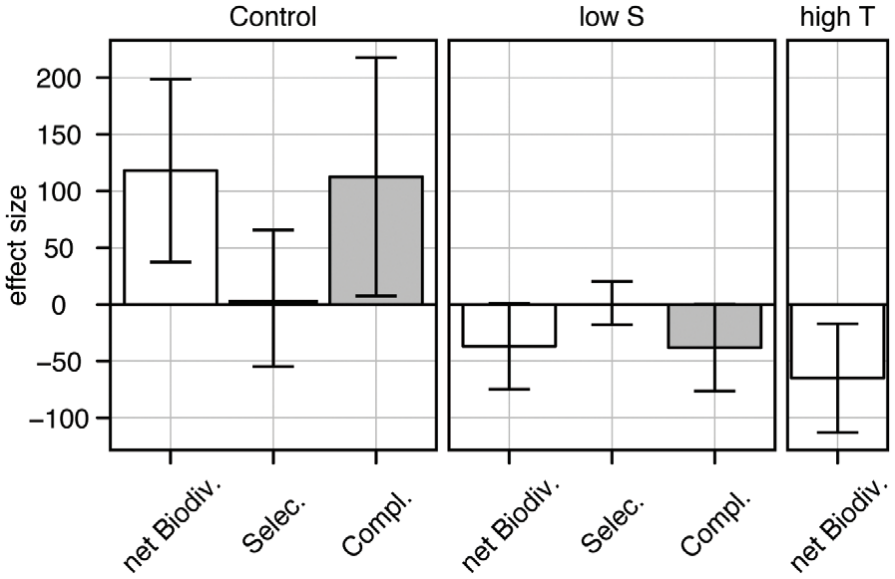
Partitioning of the diversity effect. Partitioning of the net diversity effect into a selection and a complementarity effect for the control (Salinity 25, Temperature 20°C) and the low salinity (Salinity 7, Temperature 20°C) treatments. For the high temperature treatment (Salinity 25, Temperature 28°C) the partitioning was not possible and only the net diversity effect is shown. Error bars represent the 95% confidence intervals.

The calculation of the biodiversity effects (Figure 5) showed a net positive biodiversity effect (119±76; [mean ±95% confidence interval]), a positive complementarity effect (114±103), but no selection effect (5±63) in the control treatment. In contrast, the calculation in the low salinity treatment showed a negative net biodiversity effect (–38±39, significant at the 94% confidence interval) and complementarity effect (–39±38), but again no selection effect (1.6±19) was observed. In the high temperature treatment, the net biodiversity effect was negative (–65±48) but complementarity and selection effects could not be assessed.

## Discussion

Our results showed that 1) the different genotypes were phenotypically different, 2) strong competition between the genotypes occurred, including competition mechanisms beyond outgrowing as one strain with average monoculture performance dominated the mixture, and 3) that the effects of genotypic diversity were different under control and stress conditions, but that these effects were not easily predictable from observations of the monocultures. Below, we discuss the results in more detail.

In concordance with hypothesis 1**,** the overall growth was reduced in the low salinity and high temperature treatments. The combined effect of both stressors was lethal (Figure 1). Based on the strong reaction caused by each single stressor, this was predicted based on an additive stress model (*i.e.* the sum of the impacts was expected to push biomass below 0). Additive interactions are proposed for stressors that act on indepent physiological processes which was likely the case in our experiment [38]. The physiological explanation for the 100% mortality was hence most likely the metabolic cost of each stress resistance mechanism, which exceeded the threshold of viability when both stressors were combined. This could include the activity of ion pumps to adjust the osmotic pressure under salinity stress, or the expression of heat-shock-proteins as a reaction to temperature stress. Due to the strength of each single stressor, we were unable to assess synergism (*i.e.* a greater than additive effect).

Contrary to hypothesis 2a, we found no differences in growth dynamics among the strains within the control treatment. We did, however, observe differences between strains within the high temperature and the low salinity treatment as well as in the response of at least one strain (strain 2) between the high temperature and the low salinity treatments, thereby fulfilling hypothesis 2b.

According to hypothesis 3, the most productive strain in monoculture should out-compete the other strains in the mixture and therefore become numerically dominant over time (*i.e.* positive selection effect [20]). The treatment in which we observed the greatest differences between strains in monocultures was the low salinity treatment. Strains 1 and 4 performed better than strain 2 with the highest average performance reached by strain 1 (Figure 2). The dominant strain in the mixture at the end of the experiment was strain 4 (>80%). In contrast to what was expected, the mixture performed worse than both strains 1 and 4, and we observed a negative net biodiversity effect. Strain 4 was equally dominant in the control treatment, although no differences in performance of the single strains were observed in monoculture. Moreover, in this treatment we observed a positive net biodiversity effect. Hence, even though one strain dominated in mixture, the dominant strain could not be predicted by the performances of the different strains in monoculture.

Hypothesis 4 predicted that the mixture should be more productive than the most productive monocultures (transgressive over-yielding) if interactions among strains were strongly positive and not counteracted by negative selection effects. This was not the case. A positive net biodiversity effect was observed in the control treatment, whereas the effect was negative in both stress treatments (Figure 5). We could partition the net effect of diversity into selection and complementarity effects in only two treatments (control and low salinity), and in neither of these could the net effect be attributed to a selection effect. This indicated positive complementarity in the control and negative complementarity in the low salinity treatment. Only a handful of studies have manipulated genetic diversity and partitioned the diversity effects into selection and complementarity. Positive biodiversity effects appear to be common under favourable conditions [17]–[19], [42]–[44] (but see [45]) and mostly attributed to positive complementarity [19], [42]–[44]. In contrast, among the studies that included a stress or disturbance treatment, none reported a negative net biodiversity effect, or negative complementarity [10], [18], [42], [46] although a negative selection effect was reported in three cases [10], [42], [46]. Only one study showed negative complementarity [45] and no study described a shift from positive to negative complementarity as we observed it in our experiment. This shift could be explained by the fact that both positive complementarity (e.g. resource partitioning) and negative complementarity (e.g. competition) acted simultaneously, with an increasing relative importance of negative complementarity in stressful environments. This would assume mechanisms of direct competition between the four strains, which is plausible but has not been described to date. At the species level, however, similar results were found in an experiment with different *Chladomydomonas* species [16], where non-transgressive over-yielding was observed in some nutrient-environments but not in others. It was concluded that strong but different types of interactions took place.

It has been argued that negative complementarity can be the outcome of competition among functionally similar organisms [47]. Indeed, Cadotte et al. [48] found that phylogenetic distance is the single best predictor for plant productivity in mixtures, and Jousset et al. [49] reported that genotypic richness reduces the growth rate of a bacterial community, whereas growth is enhanced by genotypic dissimilarity between community members. Likewise, in Bell’s experiment [17], mixtures composed of half-siblings outperformed mixtures composed of full-siblings. Our experiment was composed of four randomly chosen strains and we have no information on the relatedness of these strains. Although high similarity is consistent with the lack of differences between the strains under control conditions, it does not explain the positive complementarity observed in this treatment. Furthermore, phenotypical dissimilarity was higher in the high temperature and the low salinity treatment where negative complementarity was observed. In summary, we found that diversity effects occurred, but we can only speculate about the underlying mechanisms.

One of the most intriguing outcomes was the dominance of strain 4, which out-competed all other strains in the two treatments for which we had information on the final clonal composition. In a community under exponential growth with equal initial concentrations and incubation time, the only explanation for one strain becoming dominant over time is a higher growth rate. Although the growth rates of the four strains did not differ in monocultures, the relative growth rates of the individual strains in the mixture must have been different while simultaneously sustaining the overall community growth rate. Different growth rates in the mixture indicate the existence of a mechanism that affects growth rates only when strains coexist, thereby excluding classical explanations such as differences in the efficiency of resource use [50] that should equally enhance growth rates in the monocultures. Sedimentation rates were high in our experimental system and the diatoms spent most of the time at the bottom of the flasks. One possible explanation for the dominance of strain 4 in the mixture could be better access to light through a slower rate of sedimentation. In fact, strain 4 had a slightly longer average chain length than strains 1–3 (one factor ANOVA with “strain” as factor and subsequent post-hoc Tukey HSD test, *P<*0.1 in all cases). It is unclear, however, if longer chain length increases floatability in living cells [51], and stratification in only 4 cm water column seems unrealistic. A positive correlation between chain length and growth rate has also been reported [52], meaning that longer chains could actually be the effect of – and not the reason for – a competitive advantage. Another possible explanation could be chemical interference (allelopathy), which can inhibit the growth of competitors. Diatoms are known to produce toxic polyunsaturated aldehydes (PUAs) in response to cell damage [53]. Taylor et al. [54] described different production potentials of PUAs for genetically distinct strains of *S. marinoi,* and the release of PUAs without cell damage was also reported [55], yet the latter occurred only in late stationary growth phase. Although allelopathy is a well-documented phenomenon in autotrophic plankton [56], it has not been thoroughly studied at intra-specific level and an explanation for this is less clear. The compounds produced need to be extremely strain-specific, or one would need to assume a trade-off between an enhanced production of and a decreased vulnerability to the produced compounds for a specific strain. It is not clear to what extent *S. marinoi* is vulnerable to its own compounds.

In times of rapid global change, it is necessary to assess the role of genetic diversity in coping with multiple drivers of environmental shift. While the combination of stressors was lethal, we found that some algal strains grew better than others in low salinity. Only by simultaneously manipulating several stressors as well as genetic diversity can we increase our understanding of the potential importance of intraspecific biodiversity for ecosystem processes such as productivity. While we observed strong competition among strains, the mechanism behind the superiority of the dominant strain is difficult to assess. Further studies are required to evaluate the processes involved in intraspecific competition in microalgae. The study confirms, however, that many processes observed at the species level are relevant at the level of genotypes, and that genetic diversity may therefore play a comparably important role in natural systems and in changing environmental conditions.

## Supporting Information

Figure S1 **Alternative presentation of the results in** **Figure 1** **.** Growth curves of strain 1–4 (red, green, yellow and blue, respectively) and the mixture (violet). The five panels represent the four strains and the mixture and the lines represent the growth curves of the respective strain in the four treatments. Data points represent the mean of the four replicates; error bars represent standard error of mean. Biomass units are raw fluorescence data.
(TIF)
