# Supplementary figures and images for: Genetic Diversity and Ecosystem Functioning in the Face of Multiple Stressors
Source: PLoS One. 2012 Sep 18;7(9):e45007. doi: 10.1371/journal.pone.0045007 (PMC3445582; doi:10.1371/journal.pone.0045007)

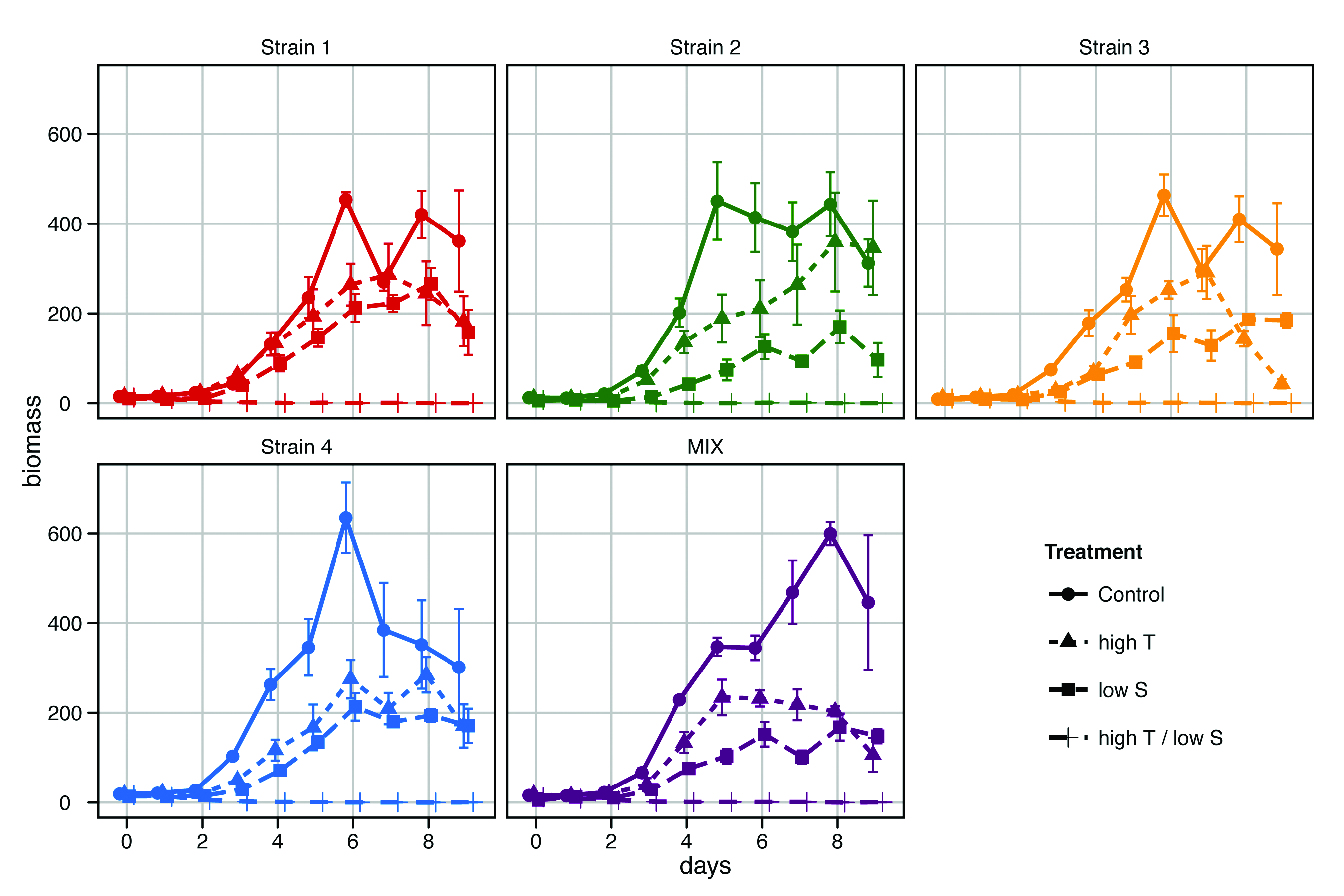

Supplement: Figure S1 — Alternative presentation of the results in Figure 1 . Growth curves of strain 1–4 (red, green, yellow and blue, respectively) and the mixture (violet). The five panels represent the four strains and the mixture and the lines represent the growth curves of the respective strain in the four treatments. Data points represent the mean of the four replicates; error bars represent standard error of mean. Biomass units are raw fluorescence data. (TIF) [file pone.0045007.s001.tif]
